# Acidic Residue Glu199 Increases SUMOylation Level of Nuclear Hormone Receptor NR5A1

**DOI:** 10.3390/ijms141122331

**Published:** 2013-11-13

**Authors:** Chiung-Min Wang, Runhua Liu, Lizhong Wang, Wei-Hsiung Yang

**Affiliations:** 1 Department of Biomedical Sciences, Mercer University School of Medicine, Savannah, GA 31404, USA; E-Mail: meowy200@yahoo.com; 2 Department of Genetics and Comprehensive Cancer Center, University of Alabama at Birmingham, Birmingham, AL 35294, USA; E-Mails: runhua@uab.edu (R.L.); lwang12@uab.edu (L.W.)

**Keywords:** NR5A1/SF1, SUMOylation, transcriptional activity, NDSM

## Abstract

Steroidogenic factor 1 (NR5A1/SF1) is a well-known master regulator in controlling adrenal and sexual development, as well as regulating numerous genes involved in adrenal and gonadal steroidogenesis. Several studies including ours have demonstrated that NR5A1 can be SUMOylated on lysine 194 (K194, the major site) and lysine 119 (K119, the minor site), and the cycle of SUMOylation regulates NR5A1’s transcriptional activity. An extended consensus negatively charged amino acid-dependent SUMOylation motif (NDSM) enhances the specificity of substrate modification by SUMO has been reported; however, the mechanism of NDSM for NR5A1 remains to be clarified. In this study, we investigated the functional significance of the acidic residue located downstream from the core consensus SUMO site of NR5A1. Here we report that E199A (glutamic acid was replaced with alanine) of NR5A1 reduced, but not completely abolished, its SUMOylation level. We next characterized the functional role of NR5A1 E199A on target gene expression and protein levels. We found that E199A alone, as well as combination with K194R, increased *Mc2r* and *Cyp19a1* reporter activities. Moreover, E199A alone as well as combination with K194R enhanced NR5A1-mediated STAR protein levels in mouse adrenocortical cancer Y1 cells. We also observed that E199A increased interaction of NR5A1 with CDK7 and SRC1. Overall, we provide the evidence that the acidic residue (E199) located downstream from the core consensus SUMO site of NR5A1 is, at least in part, required for SUMOylation of NR5A1 and for its mediated target gene and protein expression.

## 1. Introduction

Steroidogenic factor 1 (NR5A1/SF1) is a nuclear receptor and plays a crucial role in the regulation of steroid hormone biosynthesis, as well as in the endocrine development and differentiation of both the gonads and adrenal glands [1–4]. NR5A1 targets a variety of genes, including *AMH* [5], *CYP17A1* [6], *CYP19A1* [7], *DGKθ* [8], *GnRHR* [9], *GSTA3* [10], *MC2R* [11], and *STAR* [12] in humans. Regulation of these NR5A1-dependent genes mostly involves the arranged effort of NR5A1 with multiple transcription factors and cofactors with which it can coordinate and synergize, such as PITX1 [13], GATA4 [14], EGR1 [15], SOX9 [16], SREBP1 [17], and WT1 [18]. Several transcriptional co-activators, such as nuclear receptor coactivator 1 (SRC1/NCOA1) [19], cyclic AMP response element-binding protein (CREB)-binding protein/p300 [20], transcriptional intermediary factor 2 (TIF2) [21], and CTNNB1 (β-catenin) [22], have been reported to interact with NR5A1 and probably participate in NR5A1-mediated gene activation. On the other hand, transcriptional co-factors, such as DDX20 [23], nuclear receptor corepressor 1 (NCOR1) [24], and NR0B1 [25], appear to play an inhibitory role by restraining NR5A1 function. Recently, structural and functional analyses have revealed that phospholipids (such as phosphatidic acid) can functionally serve as NR5A1 ligands [26]. Recent extensive clinical studies have also found that NR5A1 is associated with birth defects and developmental disorders, such as adrenal agenesis and aplasia [27], androgen insensitivity syndrome [28], gonadal dysgenesis [29], hypospadias [30], anorchia with microphallus [31], and infertility [32]. Therefore, NR5A1 is not only critical for regulation of steroid hormone biosynthesis but also essential for endocrine organ/tissue development in adrenal glands and gonads.

Nuclear receptors are functionally regulated by post-translational modifications which are crucial for normal physiological functions in cells and efficient ways for the cells to respond to intra- and extra-cellular signals. Among post-translational modifications, the modification by small ubiquitin-related modifier (SUMO) family, a reversible modification used extensively as a regulatory mechanism in eukaryotic cells, has major effects on regulating and influencing diverse cellular pathways and processes, mainly in regulation of transcriptional activity [33–37]. Four SUMO family members (SUMO1 to −4, ranging from 90 to 110 amino acids) are encoded by distinct genes in mammals. Functional heterogeneity study has shown that the closely-related (85% identity) SUMO2 and SUMO3 have approximately 46% identity to SUMO1 [38]. In contrast to SUMO1, SUMO2, and SUMO3 possess a clear consensus SUMOylation site in their *N*-terminal regions, suggesting that SUMO2 and SUMO3 are allowed to form poly-SUMO chains [39]. Functional studies have shown that cellular stress favors SUMO2 and SUMO3 conjugations, but not SUMO1 conjugation [38]. Currently, though SUMO4 polymorphism is associated with the susceptibility of type 1 and type 2 diabetes mellitus [40], the biological role of SUMO4 remains poorly understood. In spite of limited sequence identity, SUMO proteins share with ubiquitin a common three-dimensional structure and use a similar conjugation mechanism, an enzyme-controlled pathway. Importantly, covalent conjugation of proteins by SUMO is highly transient and reversible through action of the SENP family of proteases. Even though the three-dimensional structure and conjugation pathway of SUMO share similarities to those of ubiquitin, the biological functions of SUMOylation are much different from those of ubiquitination [41]. SUMOylation mainly prevents their ubiquitin-mediated proteasomal degradation [42]. Most importantly, SUMO modification of transcription factors and nuclear receptors has a strong impact on their regulation of transcription of genes [37,43–46]. Several components of the SUMO pathway are also involved in regulation of transcription. Collectively, understanding the regulation of protein SUMOylation is important for various biological processes such as regulation of transcription and development of disorders.

Accumulated evidence indicates that post-translational modifications regulate NR5A1 functions. For example, SUMOylation at K194 inhibits and phosphorylation at S203 activates NR5A1 [47,48]. We, and others, have demonstrated that SUMOylation inhibits NR5A1 activity by reducing phosphorylation at S203 [37,49], suggesting the importance of interplay between SUMOylation and phosphorylation. Recent studies on SUMO substrates also suggest that SUMOylation is regulated by the acidic residues located downstream from the core consensus SUMO site of the target proteins [50]. However, the mechanism of the consensus negatively charged amino acid-dependent SUMOylation motif (NDSM) on NR5A1 remains to be clarified. In the present work, we demonstrate that the acidic residue (E199) located downstream from the core consensus SUMO site of NR5A1 is, at least in part, required for SUMOylation of NR5A1 and for NR5A1-mediated target gene and protein expression.

## 2. Results

### 2.1. E199A Reduces NR5A1 SUMOylation

**Figure 1:**
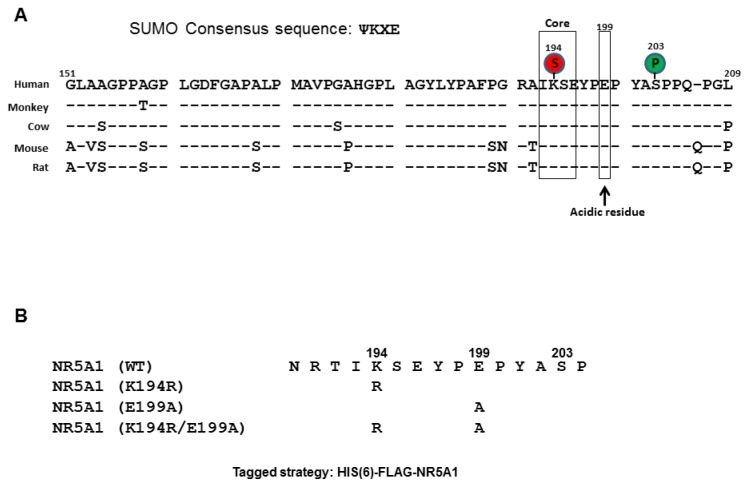
Protein sequence alignment of NR5A1 and experimental strategy. (**A**) Sequence alignment of the human, monkey, cow, mouse, and rat NR5A1 proteins showing the regions that contain the potential SUMO site (S) followed by 12–13 downstream amino acids including phosphorylation site (P); (**B**) Schematic representation of the mouse NR5A1 protein with the lysine-to-arginine and/or glutamic acid-to-alanine NR5A1 mutants generated in this study to determine potential SUMOylation sites and activities on NR5A1.

**Figure 2:**
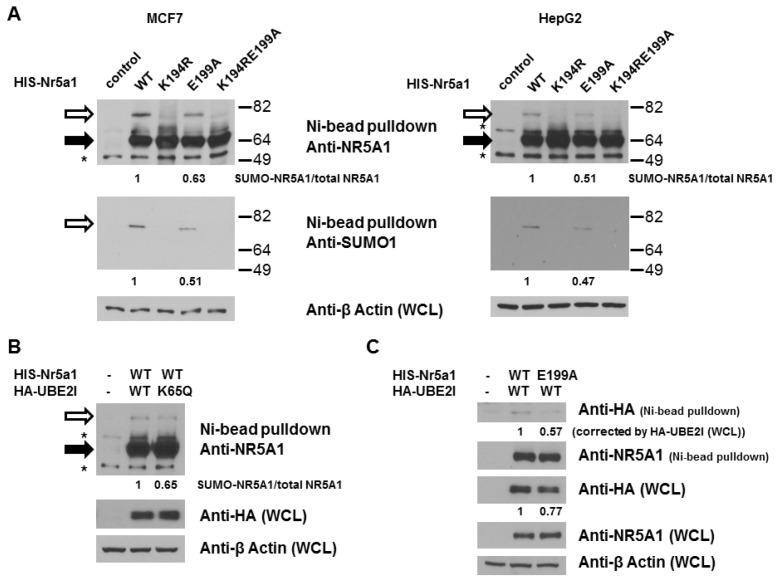
E199A reduces NR5A1 SUMOylation. (**A**) MCF7 and HepG2 cells were transiently transfected with 3 μg HIS-FLAG-tagged WT *Nr5a1* or *Nr5a1* in which lysine 194 (K194R), glutamic acid 199 (E199A), or both lysine 194 and glutamic acid 199 (K194RE199A) were mutated to arginine or alanine. Forty-eight hours later, cell lysates were subjected to Ni^2+^ bead pulldown under denaturing condition, followed by anti-NR5A1 or anti-SUMO1 immunoblotting; (**B**) HepG2 cells were transfected with 2 μg HIS-FLAG-tagged WT *Nr5a1* with or without 1μg HA-tagged *UBE2I* (WT or K65Q). Forty-eight hours later, cell lysates were subjected to Ni^2+^ bead pulldown under denaturing condition, followed by anti-NR5A1 immunoblotting; (**C**) MCF7 cells were transfected with 2 μg HIS-FLAG-tagged *Nr5a1* (WT or E199A) with or without 1μg HA-tagged *UBE2I*. Forty-eight hours later, cell lysates were subjected to Ni^2+^ bead pulldown under non-denaturing condition, followed by anti-HA or anti-NR5A1 immunoblotting. The empty arrows indicate SUMOylated NR5A1. The solid arrows indicate non-SUMOylated NR5A1. WCL indicates whole cell lysates. * indicates non-specific band. Experiments were performed three times with similar results.

NR5A1 harbors two evolutionarily conserved sequences that conform to the SUMOylation consensus: K194, the major SUMO site, and K119, the minor SUMO site. NR5A1 activity is mainly dependent on its phosphorylation at S203. We, and others, have demonstrated that SUMOylation inhibits NR5A1 activity by reducing phosphorylation at S203 [37,49], suggesting the importance of interplay between phosphorylation and SUMOylation. As NDSM has been shown to play an important role in regulation of SUMOylation and NR5A1 has one acidic residue, E199 (conserved among mammalian species), downstream from the consensus SUMO (K194) site (Figure 1A), we decided to examine the role of E199 on SUMO modification of NR5A1. To facilitate the analysis of NR5A1 SUMOylation, we created HIS-FLAG-tagged mutant forms of NR5A1 in which the acceptor K194 was replaced with arginine and E199 was replaced with alanine (Figure 1B). Importantly, these mutant forms of NR5A1 can be readily isolated and distinguished by virtue of the associated HIS and FLAG tags. To determine the role of E199 on NR5A1 SUMOylation, we probed NR5A1 preparations isolated from MCF7 and HepG2 cells by Ni^2+^ chelate chromatography under denaturing condition. As shown in Figure 2A, a slowly migrating species (about 75 kDa) was detected in cells expressing WT NR5A1. We interpret this form as being NR5A1 modified by endogenous SUMO1. Moreover, this species is not observed in cells expressing an NR5A1 form with a mutation in SUMOylation motif (K194R). This result confirms the previous studies that K194 is the major SUMO site for NR5A1 [37,47,49,51]. Interestingly, disruption of the downstream acidic residue (E199A) produced a significant reduction (37%–53% reduction compared to WT NR5A1) in NR5A1 SUMOylation. As expected, mutations of both K194 and E1991 led to a complete loss of detectable SUMOylation. Taken together, these results indicate that the acidic residue, E199, downstream from the core consensus K194 SUMO site is important in enhancing the efficiency of NR5A1 SUMOylation. A previous report has shown that UBE2I acetylation attenuates UBE2I binding to NDSM substrates, causing a reduction in NDSM substrate SUMOylation [52]. Thus, we next examined whether UBE2I acetylation is involved in NR5A1 SUMOylation. As shown in Figure 2B, a reduced SUMOylation level of NR5A1 was observed in cells expressing UBE2I K65Q, an UBE2I acetylation-mimic mutant. As the interaction between UBE2I and SUMO substrate is important for substrate SUMOylation, we next tested whether E199A mutant attenuates UBE2I binding. As shown in Figure 2C, a reduced UBE2I interaction was observed in cells expressing NR5A1 E199A mutant. These data suggest that acetylation of UBE2I at K65 reduces its interaction with NR5A1 and E199 residue is important in enhancing the efficiency of NR5A1 SUMOylation by binding to UBE2I.

### 2.2. E199A Increases NR5A1 Transcriptional Activity

**Figure 3:**
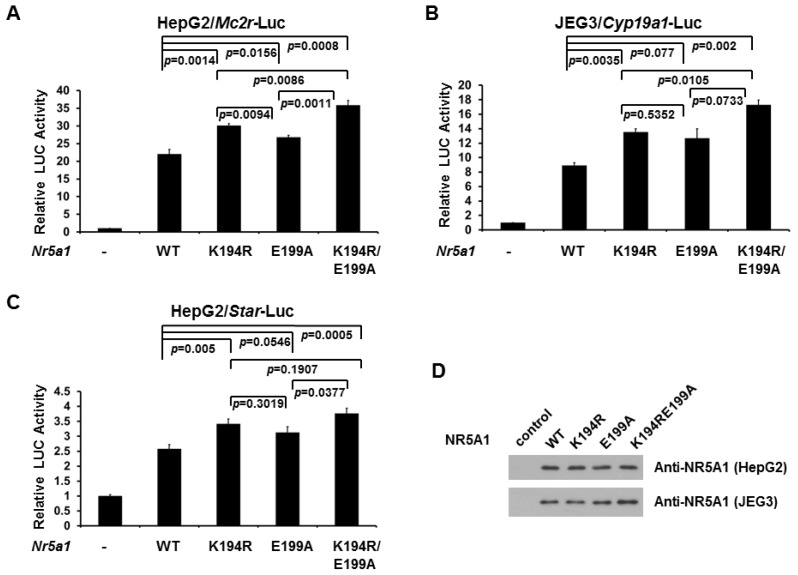
E199A increases NR5A1 transcriptional activity. (**A**) HepG2 cells were transfected, where indicated, with WT, K194R, E199A, or K194RE199A *Nr5a1* expression plasmid (0.1 μg), and a reporter plasmid with *Mc2r* natural promoter (0.1 μg); (**B**) JEG3 cells were transfected, where indicated, with WT, K194R, E199A, or K194RE199A *Nr5a1* expression plasmid (0.1 μg), and a reporter plasmid with *Cyp19a1* natural promoter (0.1 μg); (**C**) HepG2 cells were transfected, where indicated, with WT, K194R, E199A, or K194RE199A *Nr5a1* expression plasmid (0.1 μg), and a reporter plasmid with *Star* natural promoter (0.15 μg). Luciferase activities were measured at 48 h after transfection. Luciferase activity was measured and normalized with Renilla activity. Relative LUC activity (fold activation) was calculated and plotted. Experiments were performed three times in triplicate. Error bars indicate standard errors; (**D**) The expression levels of WT NR5A1 and NR5A1 mutants in HepG2 and JEG3 cells from the reporter assays (A and B, respectively) were validated using anti-NR5A1 immunoblotting.

The robust steroid hormone biosynthesis depends upon the expression of a battery of genes encoding multiple enzymes involved in steroidogenesis. As NR5A1 is a master regulator of the expression of many such genes involved in steroidogenesis, we next investigated the role of E199A NR5A1 mutant on *Mc2r* and *Cyp19a1* gene transcription by reporter gene assays. As shown in Figure 3A (HepG2 cells) and Figure 3B (JEG3 cells), expression of WT NR5A1 leads to a robust increase in the activity of a *Mc2r* and *Cyp19a1* promoter-driven luciferase reporter, respectively. As expected, disruption SUMO modification of NR5A1 (K194R) further increased the activity of *Mc2r* and *Cyp19a1* promoter-driven luciferase reporter, suggesting that SUMOylation of NR5A1 reduces its transcriptional activity. Interestingly, we observed that removal of acidic residue (E199A) downstream from the K194 SUMO site of NR5A1 also increased the activity of *Mc2r* and *Cyp19a1* promoter-driven luciferase reporter, suggesting that acidic residue located downstream from the SUMO site is essential for determining the efficiency of NR5A1 SUMOylation and hence transcriptional regulatory properties. We also observed the similar results on *Star* promoter activity in HepG2 cells (Figure 3C). The expression levels of WT NR5A1 and NR5A1 mutants in HepG2 and JEG3 cells from the reporter assays were validated in Figure 3D. These findings indicate that the acidic residue (E199) located downstream from the core consensus K194 SUMO site of NR5A1 is, at least in part, required for SUMOylation of NR5A1 and for NR5A1-mediated target gene expression.

### 2.3. E199A Increases NR5A1’s Interaction with CDK7 and SRC1 as well as STAR Protein Level

**Figure 4:**
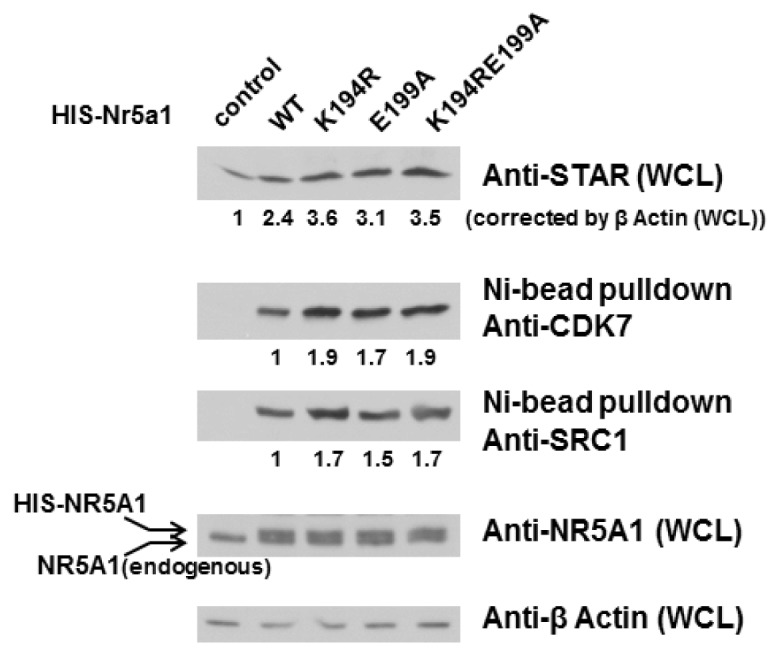
E199A increases NR5A1’s interaction with CDK7 and SRC1 as well as STAR protein level. Mouse adrenocortical cancer Y1 cells were transfected with WT, K194R, E199A, or K194RE199A *Nr5a1* expression plasmid (1 μg). After 48 h, whole cell lysates (WCLs) were collected and then subjected to Ni^2+^ bead pulldown, followed by anti-CDK7 or anti-SRC1 immunoblotting. WCLs were validated using anti-STAR immunoblotting to measure STAR protein levels. The expression levels of NR5A1 and β-Actin in WCLs were also validated using anti-NR5A1 and β-Actin immunoblotting, respectively. Experiments were performed three times with similar results.

We and others have previously shown that NR5A1 S203 phosphorylation is mediated by CDK7. Since CDK7 interacts with NR5A1 and CDK7 inhibitors block both phosphorylation of NR5A1 and its transactivation capacity, we next investigated whether E199A alters the interaction of NR5A1 and CDK7. We therefore used Y1 cells expressing WT or mutant NR5A1 to examine NR5A1-associated proteins. As shown in Figure 4, NR5A1 preparations isolated via Ni^2+^ chelate chromatography under nondenaturing condition contained larger amount of associated CDK7 in the case of the mutant NR5A1 than in that of WT NR5A1 (1.9- and 1.7-fold for the K194R and E199A forms of NR5A1, respectively). However, the combination of K194R and E199A did not further increase the interaction of NR5A1 and CDK7. We also detected increased recovery of the coactivator SRC1 related to WT NR5A1 (1.7- and 1.5-fold for the K194R and E199A forms of NR5A1, respectively). However, again, the combination of K194R and E199A did not further increase the interaction of NR5A1 and SRC1. This indicates that loss of acidic residue downstream from the K194 SUMO site of NR5A1 facilitates its interaction with CDK7 and SRC1. As the expression of STAR protein, the rate-limiting factor in the steroidogenesis pathway, is directly regulated by NR5A1 in adrenal glands and gonads, we next examined whether loss of acidic residue resulted in greater expression of STAR proteins. As depicted in Figure 4, as expected and consistent with the previous reports [37], STAR protein levels were indeed increased in K194R NR5A1 expressing Y1 cells. Interestingly, STAR protein levels were also increased in E199A NR5A1 expressing Y1 cells, suggesting that loss of acidic residue not only reduces NR5A1 SUMOylation but also enhances the expression levels (promoter activities and protein levels) of the enzymes involved in steroidogenesis. Taken together, these results provide evidence that the acidic residue downstream from the core K194 SUMO site of NR5A1 is essential, at least in part, for NR5A1 SUMOylation and interaction with others.

## 3. Discussion

Regulation of protein function by reversible post-translational modifications is the core principle in cell biology and biochemistry. Modifications with SUMO family of proteins have emerged as essential and critical events in a variety of biological processes, including apoptosis, cell cycle regulation, cell growth and differentiation, genomic instability, transcriptional regulation, tumorigensis, and protein-protein interactions. SUMO modification of NR5A1 has a strong influence on its transcriptional activity as evidenced by that NR5A1 transcriptional activity is intensively inhibited by SUMOylation [37,47,51]. Though an extended NDSM enhances the specificity of substrate modification by SUMO has been reported previously [50]; however, the functional significance of NDSM for NR5A1 remains to be clarified. In the present work, we demonstrate that the acidic residue downstream from the core SUMO consensus motif of NR5A1 is essential, at least in part, for NR5A1 SUMOylation and NR5A1-mediated transcriptional activity.

While hundreds of SUMO-conjugated substrates have been identified, the precise mechanisms that differentially regulate substrate SUMOylation are still largely unknown. We also know little about the mechanisms how a specific lysine residue in a SUMO-conjugated substrate which has more than one SUMO site is being preferentially attached by SUMO. The majority of discovered SUMO substrates are recognized by SUMO E2 conjugase UBE2I (also called UBC9) to a consensus motif ΨKxE (where Ψ stands for a large hydrophobic amino acid, such as V, I, L, and F). A recent study on site-specific identification of SUMO2 targets revealed that 76 out of 103 SUMO2 acceptor lysines in endogenous target proteins are situated in the SUMOylation consensus site (approximately 76%) [53]. Moreover, several lines of evidence recently suggest that amino acids upstream or downstream from the SUMOylation consensus tetrapeptide also facilitate substrate SUMOylation. For example, phosphorylation-dependent SUMOylation motif (PDSM) and negatively charged amino acid-dependent SUMOylation motif (NDSM) contain additional phosphorylated and negatively charged amino acids sequences downstream to the consensus motif, respectively. Another example is that a hydrophobic cluster SUMOylation motif (HCSM) contains a series of hydrophobic amino acids (such as V or I) upstream of the consensus motif [53]. In NDSM’s case, the negatively charged amino acids (acidic residues) downstream from the consensus motif are thought to dramatically enhance UBE2I interaction (by binding to a basic patch on UBE2I) and subsequently increase SUMOylation process. We have previously shown that while phosphorylation does not influences its SUMOylation, NR5A1 SUMOylation reduces it phosphorylation by reducing CDK7 interaction [37], suggesting that PDSM is not participated in NR5A1 SUMOylation. Several lines of evidence have demonstrated that numerous UBE2I-interacting proteins are SUMOylated including NR5A1 [54,55], suggesting NDSM and/or HCSM might be present in NR5A1. While we could not find HCSM in NR5A1 based on its protein sequence, we indeed found E199 downstream from the core K194 SUMO site in NR5A1. In the present study, we demonstrated that E199 is essential for NR5A1 SUMOylation because mutation of E199 reduces NR5A1’s SUMOylation (Figure 2), suggesting its role in enhancing UBE2I interaction and subsequently facilitating NR5A1 SUMOylation (Figure 2B,C). Since SUMOylation of NR5A1 reduces its transcriptional activity on target genes, the results of the reporter gene assays from the current study (Figure 3) further support that E199 is involved and essential, at least in part, for NR5A1 SUMOylation, demonstrating that the importance of acidic residues downstream the core consensus SUMO site on NR5A1.

NR5A1-mediated gene transcription is essential for steroidogenesis as well as adrenal and gonadal development and differentiation. For example, binding of cofactors such as SRC1 and CBP to NR5A1 is necessary to fully activate NR5A1-mediated CYP17 transcription [56]. Previously, we have shown that CDK7 and SRC1 bind preferentially to the SUMOylation-deficient form of NR5A1 and that CDK inhibition reduces NR5A1 phosphorylation [37], suggesting that SUMOylation inhibits NR5A1 activity by reducing CDK7-mediated phosphorylation of NR5A1. In current study, we observed that removal of acidic residue (E199A) not only reduces its SUMOylation but also increases NR5A1’s binding to SRC1 and CDK7, suggesting that E199 plays an important role in facilitating NR5A1 SUMOylation. Collectively, our results not only extend the conclusion with the previous NDSM report that acidic residues located downstream from the consensus SUMO conjugation site increase substrate SUMOylation possibly by enhancing UBE2I interaction but also provide the novel mechanism that how NDSM regulates NR5A1 activity by influencing its SUMOylation.

## 4. Experimental Section

### 4.1. Reagents

All cell culture reagents were purchased from Invitrogen (Carlsbad, CA, USA). Protein A and protein G magnetic beads were purchased from Fisher Scientific (Pittsburgh, PA, USA). Antibodies against NR5A1 (Upstate Biochemistry Inc., Charlottesville, VA, USA), STAR, CDK7, HA, and SRC1 (Santa Cruz Biotechnology Inc., Santa Cruz, CA, USA), SUMO1 (Active motif, Carsbad, CA, USA), β-Actin (Sigma, St. Louis, MO, USA) were purchased commercially. Luciferase activity was measured using the Dual Luciferase Assay System (Promega, Madison, WI, USA). Ni-NTA agarose was purchased from QIAGEN (Valencia, CA, USA).

### 4.2. DNA Constructs

Mouse *Nr5a1*-pcDNA3.1(+) expression plasmid, mouse *mc2r* promoter-LUC plasmid, and mouse *Star* promoter-LUC plasmid were previously established in our laboratory as described in Yang *et al*. [57]. K194R *Nr5a1*, E199A *Nr5a1*, and K194RE199A *Nr5a1* expression plasmids were generated by PCR-based mutagenesis (QuikChange Lightning site-directed mutagenesis kit, Strategene, La Jolla, CA, USA). The mouse *Cyp19a1* promoter (~600-bp) was PCR amplified by using forward primer 5′-ACGAGGTACCGTCCTAGTTCTAG-3′ and reverse primer 5′-TCGTAAGCTTGTTCTATGGGAAG-3′, digested with KpnI and HindIII and then ligated into the KpnI and HindIII sites of pGL3 to create *Cyp19a1* LUC plasmid. Human HA-tagged *UBE2I*-pcDNA3.1(+) expression plasmid was previously established in our laboratory as described in Wang *et al*. [35]. K65Q HA-*UBE2I* expression plasmid was generated by PCR-based mutagenesis (QuikChange Lightning site-directed mutagenesis kit, Strategene, La Jolla, CA, USA). All constructs were verified by nucleotide sequencing.

### 4.3. Cell Culture and Transfection

HepG2, JEG3, MCF7, and Y1 cells were purchased from the American Type Culture Collection. HepG2, MCF7, and JEG3 cells were maintained in Dulbecco’s modified Eagle’s medium (DMEM) in the presence of 10% fetal bovine serum and antibiotics (GIBCO/Life Technologies, Grand Island, NY, USA) in humidified air containing 5% CO_2_, at 37 °C. Y1 cells were maintained in DMEM supplemented with 7.5% horse serum and 2.5% fetal bovine serum and antibiotics in humidified air containing 5% CO_2_, at 37 °C. After incubation, the cells were transfected using Fugene HD Transfection Reagent (Roche, Madison, WI, USA). Approximately 45−48 h after transfection, the cells were harvested. Luciferase activity was measured and normalized with Renilla activity. All experiments were performed three times in triplicate.

### 4.4. Immunoprecipitation Assay

MCF7, HepG2 or Y1 cells (2 × 10^6^) were seeded onto 10-cm plates. Twenty-four hours after transient transfection, cells were harvested and lysed in lysis buffer (40 mM HEPES, 120 mM sodium chloride, 10 mM sodium pyrophosphate, 10 mM sodium glycerophosphate, 1 mM EDTA, 50 mM sodium fluoride, 0.5 mM sodium orthovanadate, 1% Triton X-100) containing protease inhibitor cocktail (Sigma), followed by rotation for 1 h at 4 °C to solubilize proteins. Soluble protein was collected and immunoprecipitated with the indicated antibody overnight. Protein A or G magnetic beads were added to protein lysates for 2 h in the cold room. Beads were separated from lysate solution by magnetic force (in a magnetic separation rack) and washed at least three times with lysis buffer. For Ni^2+^-bead pull-down assays, Ni^2+^-NTA agarose was used to precipitate HIS-tagged NR5A1 from cell lysates. Proteins were eluted by boiling in 50 μL of 2× Laemmli sample buffer, resolved by 8% SDS-PAGE, and processed for immunoblotting as described below.

### 4.5. Immunoblotting

Protein lysates were allowed to rotate at 4 °C for 30 min, and protein contents of the high-speed supernatant were determined using the BCA™ Protein Assay kit assay (Pierce/Thermo Scientific, Rockford, IL, USA). Equivalent quantities of protein (20–40 μg) were resolved on polyacrylamide-SDS gels, transferred to nitrocellulose membrane (Bio-Rad, Hercules, CA, USA), and immunoblotted with specific antibodies. Results were visualized using the Supersignal West Dura Extended Duration Substrate kit (Pierce Chemical Co., Rockford, IL, USA). Band intensity was quantified by ImageJ program.

### 4.6. *In Vivo* SUMOylation Assays

The *in vivo* SUMOylation assay was carried out as previously described [37]. Briefly, MCF7 or HepG2 cells (2 × 10^6^) were seeded in 10 cm plates and 24 h later were transfected with indicated HIS-FLAG-*Nr5a1* expression vectors. After 48 h, cells were harvested in 700 μL lysis buffer (500 mM NaCl, 10 mM imidazole, 45 mM Na_2_HPO_4_, 5 mM Na_2_H2PO_4_, 8 M urea, pH 8.0) containing complete protease inhibitors without EDTA (1 tablet/10 mL; Roche, Madison, WI, USA) and sonicated. Lysates were cleared and incubated with 100 μL of 50% Ni^2+^-NTA agarose (QIAGEN, Valencia, CA, USA) at room temperature for 60 min on a rotator. The resin was washed 3 times in wash buffer 1 (400 mM NaCl, 10 mM imidazole, 17.6 mM Na_2_HPO_4_, 32.4 mM Na_2_H_2_PO_4_, 8 M urea, pH 6.75), washed 3 times in wash buffer 2 (150 mM NaCl, 10 mM imidazole, 17.6 mM Na_2_HPO_4_, 32.4 mM Na_2_H_2_PO_4_, pH 6.75). Samples were resuspended in 2X EDTA SDS-PAGE sample buffer. Samples (20 μL) were resolved by 8% SDS-PAGE and processed for immunoblotting using anti-NR5A1 or anti-SUMO1 primary antibody. Images were captured in a Kodak Image Station 440 CF using Super Signal West Femto substrates (Thermo scientific/Pierce, Rockford, IL, USA).

### 4.7. Statistical Analysis

Statistical analyses were performed using the Student’s *t* test or a one-way ANOVA when more than two groups were compared. After the ANOVA analysis, the *post hoc* multiple comparisons were performed by using Tukey honestly significant difference (HSD) test to determine the statistical difference from each other among subgroups. For each test, *p* values less than 0.05 were considered significant.

## 5. Conclusions

In summary, this investigation has demonstrated that NDSM serves an important additional determinant of SUMO modification on NR5A1. Our study also adds a new layer to the previous understanding of how NR5A1 functions to regulate adrenal and gonadal development and differentiation as well as steroid hormone biosynthesis.

## Abbreviations

AMH: muellerian-inhibiting factor
CDK7: cyclin-dependent kinase 7
CYP17A1: steroid 17-alpha-hydroxylase/17,20-lyase
CYP19A1: cytochrome p450 19A1
DDX20: ATP-dependent RNA helicase DDX20
DGKθ: diacylglycerol kinase theta
EGR1: early growth response protein 1
GATA4: transcription factor GATA-4
GnRHR: gonadotropin-releasing hormone receptor
GSTA3: glutathione *S*-tranferase A3
MC2R: adrenocorticotropic hormone receptor
NCOR1: nuclear receptor corepressor 1
NDSM: negatively charged amino acid-dependent SUMOylation motif
NR0B1/DAX1: nuclear receptor subfamily 0 group B member 1
NR5A1: steroidogenic factor-1
PDSM: phosphorylation-dependent SUMOylation motif
PITX1: pituitary homeobox 1
SOX9: transcription factor SOX-9
SRC1/NCOA1: nuclear receptor coactivator 1
SREBP1: sterol regulatory element-binding protein 1
STAR: steroidogenic acute regulatory protein, mitochondrial
SUMO: small ubiquitin-like modifier
TIF2: nuclear receptor coactivator 2
UBE2I: SUMO-conjugating enzyme UBC9
WT1: Wilms tumor protein.
